# Multi-Target Antitumor Effects of Natural Products and Approved Drug Repurposing in Non-Small Cell Lung Cancer: Advances in Mechanisms, Combination Regimens, Delivery System Optimization, and Clinical Challenges

**DOI:** 10.3390/ijms27177908

**Published:** 2026-09-04

**Authors:** Yuli Xie, Dashuai Zhang, Pei Tang

**Affiliations:** West China School of Pharmacy, Sichuan University, Chengdu 610041, China; xieyl@scu.edu.cn (Y.X.); zhangds813@126.com (D.Z.)

**Keywords:** lung cancer, natural products, drug repositioning, combination therapy

## Abstract

Non-small cell lung cancer is one of the leading causes of cancer-related mortality. The effectiveness of treatment is limited by several factors, including significant tumoral heterogeneity, rapid development of drug resistance, high metastatic potential, and a complex tumor microenvironment. In recent years, natural products have emerged as valuable resources for the discovery of novel antitumor strategies against non-small cell lung cancer. These compounds possess diverse chemical properties, target multiple pathways, are abundant in nature, and exhibit relatively low toxicity. The utilization of existing medications with established pharmacokinetic profiles and safety records, combined with shorter development timelines, shows promise for advancing lung cancer treatment research. A growing body of evidence indicates that both naturally occurring compounds and commercially available drugs exert effects that extend beyond traditional cytotoxic mechanisms. These agents influence processes such as ferroptosis, oxidative stress, metabolic reprogramming, autophagy, apoptosis, epithelial–mesenchymal transition (EMT), tumor immune microenvironments, and epigenetic networks, suggesting that their activities can be leveraged for a robust multi-target antitumor strategy. Accordingly, this review summarizes research advances on natural products and repurposed marketed drugs for non-small cell lung cancer; outlines their potential for combination with chemotherapy, targeted therapy, radiotherapy and immunotherapy; and discusses future directions for clinical translation.

## 1. Introduction

Lung cancer remains one of the malignant tumors with the highest incidence and mortality rates worldwide, with non-small cell lung cancer (NSCLC) accounting for approximately 85% of all cases, thereby continuing to pose a significant public health burden [1,2]. In recent years, the rapid development of targeted therapy and immunotherapy has markedly improved survival outcomes for some NSCLC patients, marking a transition in the treatment of advanced lung cancer from traditional chemotherapy to precision therapy [3,4,5,6]. However, the clinical benefits of current therapeutic strategies remain limited, as issues such as diminished efficacy, acquired resistance, recurrence, metastasis following disease progression, and associated toxicities continue to represent core challenges that hinder long-term disease control [6,7,8,9]. Further studies have revealed that NSCLC exhibits considerable heterogeneity concerning driver genes, tumor immune microenvironment, and metabolic status [10,11,12]. There are substantial differences in sensitivity and tolerance to identical treatment regimens among different patients, which serves as an important biological basis for the instability of therapeutic efficacy and the evolution of drug resistance [13,14,15].

Investigating the fundamental molecular processes and the microenvironmental features of NSCLC while developing more targeted treatment approaches based on these insights not only clarify the critical mechanisms underlying tumor growth and resistance to therapy, but also offer a substantial theoretical foundation and justification for refining patient classification, boosting long-term disease management outcomes, and increasing clinical advantages [16,17].

In this context, the utilization of natural products and the repurposing of approved drugs have emerged as significant complementary strategies in the development of lung cancer therapeutics [18,19,20,21]. Natural products are distinguished by their diverse sources, rich structural frameworks, and multi-target regulatory capabilities [22,23,24]. Existing studies have demonstrated that certain natural products can inhibit tumor growth by inducing ferroptosis in NSCLC and lung adenocarcinoma, while also affecting the proliferation, migration, and invasion of tumor cells [25,26]. In contrast to the development of novel molecules, the repurposing of approved drugs capitalizes on established safety profiles, pharmacokinetic data, and prior clinical experiences to identify new indications for lung cancer within a reduced timeframe [27,28]. This approach not only mitigates development risks but also enhances translational efficiency [25,29,30].

In recent years, the research focus on tumor cell death modalities has expanded from traditional apoptosis to include regulated cell death patterns, such as ferroptosis, autophagy-dependent death, and necroptosis [31,32,33]. The interplay among these mechanisms has opened new avenues for understanding drug resistance in lung cancer and identifying strategies for sensitization [34,35,36]. In the context of lung cancer, ferroptosis is closely associated with treatment resistance, immune response, and metabolic vulnerability [37,38]. The interactions between autophagy and ferroptosis, as well as the disruption of mitochondrial homeostasis, have been repeatedly shown to influence the sensitivity of tumor cells to therapeutic agents [19,39,40]. Numerous studies indicate that natural products and conventional drugs not only exert their effects by directly killing tumor cells, but can also induce ferroptosis by perturbing lipid peroxidation, glutathione metabolism, iron homeostasis, and mitochondrial function [41,42]. Additionally, these compounds can activate signaling pathways such as AMPK/mTOR, PI3K/AKT/mTOR, and Nrf2, which further modulate the cell cycle, autophagy, apoptosis, and tumor immunity [43,44,45].

**Figure 1:**
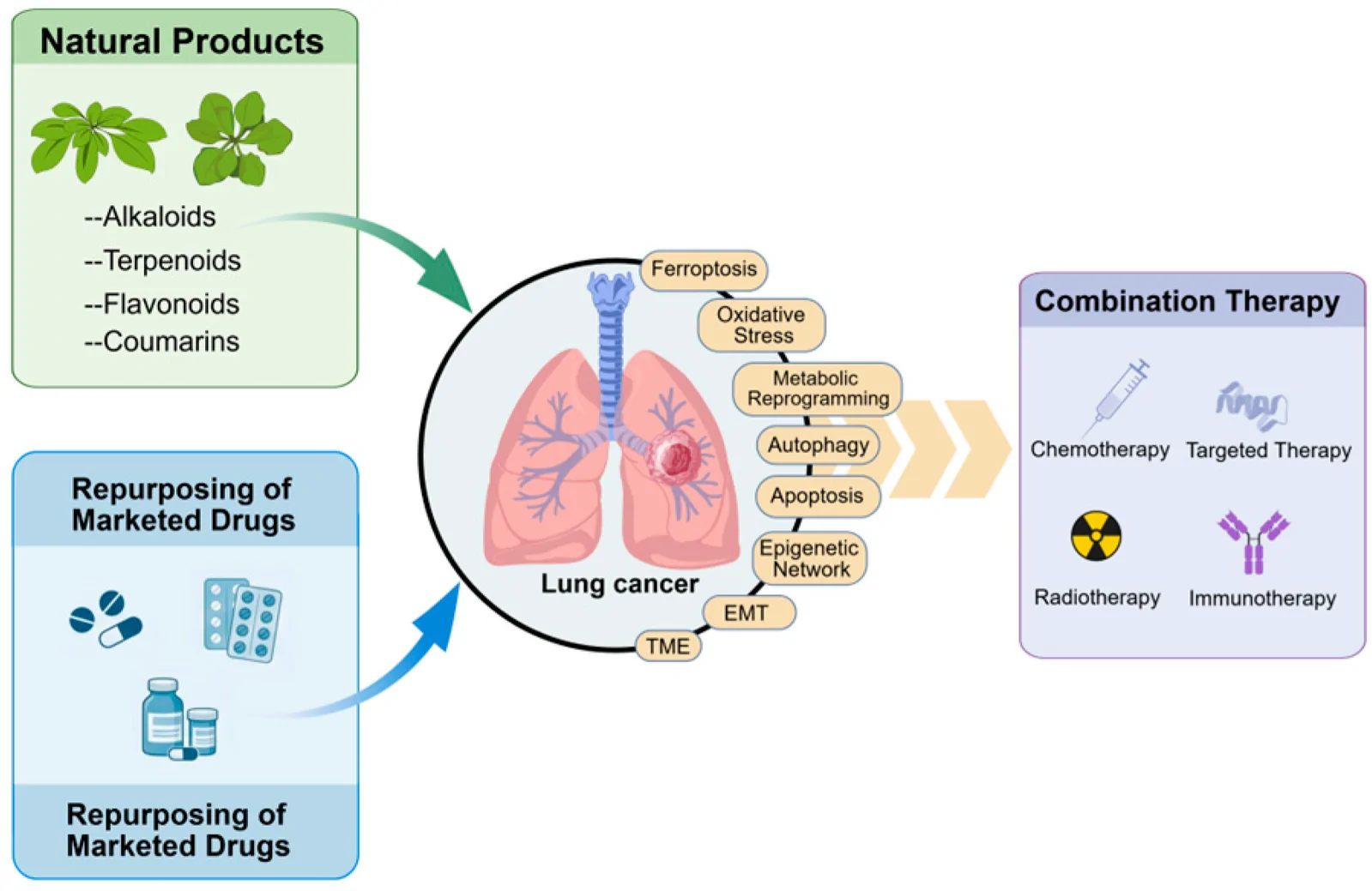
Overview diagram of the main content of the review.

Beginning with the concept of “multi-target antitumor” and progressing to “combination sensitization” and “clinical translation”, this review examines the research advancements regarding natural products and drug repositioning in non-small cell lung cancer [46,47]. This approach not only facilitates the summarization of their reproducible mechanisms of action but also aids in the identification of candidate molecules with significant development potential [19,20,48]. The review aims to delineate the challenges associated with lung cancer treatment, notable studies on natural products and the repurposing of approved drugs and strategies for combination therapy (Figure 1). The ultimate objective is to provide valuable references for subsequent basic research and clinical translation [39,49]. Furthermore, such studies may offer new insights for addressing critical clinical challenges, including drug resistance, relapse, and metastasis [35,40,50].

## 2. The Biological Basis and Research Entry Points of Lung Cancer Treatment

### 2.1. Classical Oncogenic Signaling Pathways and Therapeutic Tolerance

Common abnormal signaling networks in lung cancer include the PI3K/AKT/mTOR, MAPK/ERK, STAT3, Wnt/β-catenin, TGF-β, NF-κB, and Nrf2 pathways [51,52,53]. The distribution of these signaling pathways is shown in Table 1. The PI3K/AKT/mTOR pathway is closely linked to cell growth, protein synthesis, and metabolic maintenance [54,55,56,57,58,59]; the MAPK/ERK pathway is implicated in proliferation and survival signaling, while STAT3 is associated with inflammation, immunosuppression, and anti-apoptotic phenotypes [51,52,60]; Wnt/β-catenin, TGF-β, and NF-κB are commonly involved in epithelial–mesenchymal transition (EMT), invasion/metastasis, and therapy resistance [53,60]; and Nrf2 serves as a key transcription factor that regulates cellular antioxidant defense and redox homeostasis [61,62], but it exhibits a dual role in tumors [43,63,64]. Numerous preclinical studies have indicated that these pathways represent common targets for natural active ingredients in the intervention of lung cancer [52,65,66].

### 2.2. Classification, Heterogeneity, and Therapeutic Challenges of Lung Cancer

Lung cancer is primarily classified into two major categories: non-small cell lung cancer (NSCLC) and small cell lung cancer (SCLC) [67,68]. Among these, NSCLC predominantly encompasses lung adenocarcinoma, lung squamous cell carcinoma, and several other subtypes [69,70]. Different subtypes exhibit significant heterogeneity in driver gene profiles, immune microenvironments, metastatic propensity, and therapeutic sensitivity [71,72]. Driver gene alterations, such as EGFR, ALK, ROS1, KRAS, BRAF, and MET, present important opportunities for targeted therapy [73,74]; however, the applicable patient population is limited, and the development of acquired resistance is nearly inevitable [67,75].

The development of drug resistance in lung cancer involves multiple mechanisms, including secondary mutations in the target and the activation of bypass pathways [76,77]. Additionally, epithelial–mesenchymal transition (EMT), lineage plasticity, upregulation of DNA repair mechanisms, and the enhancement of cancer stemness are also prevalent [78,79]. Under continuous therapeutic pressure, tumor cells can sustain survival by enhancing antioxidant defenses and remodeling metabolic–redox homeostasis [77,80,81]. Immune escape is particularly common in the context of immunotherapy resistance [82,83]. Consequently, single-target or single-agent strategies frequently fail to achieve long-term efficacy [84,85]. Therefore, the primary goal of this review is to delay the emergence of this drug resistance mechanism from the perspective of combined drug administration by reviewing recent research progress.

## 3. Antitumor Effects of Natural Products in Lung Cancer

### 3.1. Research Advantages of Natural Products

Natural products serve as a pivotal source for the discovery of antitumor drugs. Historically, numerous classic anticancer agents have been derived from natural products or their derivatives [86,87,88]. In contrast to single-target synthetic drugs, natural products frequently exhibit more complex chemical structures and a wider array of biological activities, allowing them to exert a more comprehensive influence on various aspects of tumor initiation and progression. Specifically, the specific advantages and disadvantages of antitumor natural products compared with single-target antitumor drugs are shown in Table 2. Specifically for lung cancer, natural products are particularly effective in the following mechanisms of action: inducing programmed cell death, inhibiting tumor metabolic adaptation, suppressing migration and invasion, remodeling the tumor microenvironment, and enhancing sensitivity to radiotherapy, chemotherapy, and immunotherapy [89,90].

### 3.2. Anticancer Mechanisms of Natural Products

#### 3.2.1. Induction of Apoptosis

Apoptosis evasion is a key reason for the sustained survival and treatment resistance of lung cancer cells. Natural products can promote programmed cell death in lung cancer cells by activating the mitochondrial pathway, death receptor pathway, and caspase cascade, thereby inhibiting tumor growth [91,92,93,94]. Additionally, this mechanism helps enhance the sensitivity of lung cancer cells to chemotherapy, radiotherapy, and targeted therapy, reducing the survival ability of residual cells after treatment [92,93,94,95,96].

#### 3.2.2. Induced Autophagy and Cell Cycle Arrest

Lung cancer cells rely on abnormally active proliferation cycles and stress adaptation capabilities to sustain malignant growth [56,59]. Natural products can regulate autophagy levels, induce cell cycle arrest, and block DNA replication and cell division processes, thereby inhibiting tumor proliferation [97,98]. This effect may also weaken the adaptability of lung cancer cells to the stress of chemotherapy and targeted therapy, enhancing the efficacy of combination therapy [99,100,101].

#### 3.2.3. Inhibition of Migration, Invasion, and EMT

Invasion and distant metastasis are important causes of poor prognosis and treatment failure in lung cancer [102,103,104,105]. Natural products can reduce the invasive and metastatic potential of lung cancer cells by inhibiting EMT, decreasing cell motility, and impairing the ability to degrade the extracellular matrix [102,103,104,106]. This mechanism not only helps control disease progression but may also reduce the risk of tumor recurrence and metastasis following radiotherapy, chemotherapy, or targeted therapy [102,103,104,106].

#### 3.2.4. Intervening in the Tumor Microenvironment

The tumor microenvironment provides lung cancer cells with immune evasion, angiogenesis, and drug resistance support [107,108]. Natural products can modulate immune cell functions, inflammatory cytokine release, angiogenesis, and extracellular matrix remodeling, thereby undermining the survival basis of lung cancer cells. This effect helps improve the immunosuppressive state and may enhance the overall efficacy of immunotherapy, chemotherapy, and radiotherapy [109,110,111,112].

#### 3.2.5. Regulating Oxidative Stress and Mitochondrial Function

Lung cancer cells often exist in a state of abnormal oxidative stress and rely on mitochondria to maintain energy metabolism and resistance to cell death [8,113]. Natural products can disrupt redox balance, induce mitochondrial dysfunction, and promote cell death [113,114]. Since radiotherapy and certain chemotherapeutic agents are also closely associated with oxidative damage, this mechanism may enhance the sensitivity of lung cancer cells to radiotherapy and chemotherapy [19,113,115,116,117,118,119,120].

#### 3.2.6. Induction of Ferroptosis

Ferroptosis is an iron-dependent, regulated form of cell death driven by lipid peroxidation, and it plays a critical role in NSCLC pathogenesis, therapeutic response, and drug resistance. Its execution mainly involves iron metabolism, lipid peroxidation, and the SLC7A11-GSH-GPX4 antioxidant axis [19,115,121]. Elevated intracellular free Fe^2+^ accelerates lipid radical reactions, and excessive peroxidation of polyunsaturated fatty-acid-containing phospholipids causes plasma membrane damage, which serves as the direct effector process of ferroptosis. Existing evidence demonstrates that in multiple NSCLC models, including A549, H460, H1299 and PC-9, inhibition of SLC7A11 disrupts cystine uptake, reduces GSH synthesis, and impairs GPX4-mediated clearance of lipid peroxides. These events consequently trigger ferroptosis and suppress lung cancer cell proliferation [115,116,117]. Accordingly, high expression of SLC7A11 or GPX4 represses ferroptosis and may support NSCLC cell survival and therapeutic tolerance; inhibition of this axis augments lipid peroxidation and ferroptosis. Therefore, targeting iron metabolism, lipid peroxidation, or the SLC7A11-GPX4 axis may sensitize NSCLC to chemotherapy, targeted therapy, radiotherapy and immunotherapy. Nevertheless, such therapeutic strategies remain largely confined to preclinical or early translational research stages [19,115,120].

## 4. Research Progress on the Repurposing of Marketed Drugs in Lung Cancer

### 4.1. Value of Drug Repositioning

The primary advantage of drug repositioning lies in the established foundation of human safety, pharmacokinetics, and toxicology associated with existing medications. This generally leads to higher translational efficiency and shorter development cycles [122,123]. In comparison to entirely novel compounds, marketed drugs are more likely to progress to clinical reevaluation. For diseases such as lung cancer, which necessitate long-term treatment and often depend on combination regimens, drug repurposing is particularly advantageous for developing sensitizers, adjuvant therapies, or synergistic repositioning candidates within combination therapy. Nonetheless, it is crucial to reassess the dosage, toxicity, and drug interactions of older medications when applied to new indications [124,125,126].

### 4.2. Repositioning of Metabolic-Related Drugs

Metformin is one of the most representative drugs in drug repurposing research. Originally used to treat type 2 diabetes, it has been shown to reshape energy metabolism in tumors and limit tumor cell growth by inhibiting mitochondrial complex I, reducing oxidative phosphorylation, activating AMPK, and downregulating mTOR signaling [127,128]. In the context of lung cancer, metformin not only has the potential to inhibit proliferation but also demonstrates synergistic effects when combined with chemotherapy, targeted therapy, or immunotherapy in preclinical and some early-stage clinical studies. However, existing randomized clinical evidence remains inconsistent, particularly in non-small cell lung cancer (NSCLC), where no stable conclusion regarding its benefits has yet been established [129,130]. Statins inhibit HMG-CoA reductase, interfere with the mevalonate pathway, and affect cholesterol synthesis and the stability of lipid rafts in the membrane [131]. This disruption impacts tumor cell signal transduction and membrane receptor function. Due to their role in cholesterol metabolism and the structural stability of membranes, statins are also considered to have potential anti-lung cancer effects [132,133].

### 4.3. Repurposing Anti-Infective Drugs

Anti-infection-related marketed drugs represent a significant source for the repositioning of existing drugs in the treatment of lung cancer. Notably, chloroquine/hydroxychloroquine, itraconazole, artemisinin derivatives, and disulfiram have emerged as candidate drugs frequently explored in oncology research [134,135,136]. Among these, chloroquine and hydroxychloroquine are well-known for their mechanism of action, which involves the inhibition of lysosomal acidification and the blockade of autophagy flux. This action compromises the ability of tumor cells to adapt and survive under therapeutic stress [134,137]. Furthermore, itraconazole, beyond its antifungal properties, has been reported to exhibit antitumor activity and has the potential to reverse P-glycoprotein-mediated chemotherapy resistance in certain studies [8,136]. The relationship between artemisinin and its derivatives and reactive oxygen species (ROS) amplification, mitochondrial damage, and the induction of ferroptosis is well established. When combined with other sensitization strategies, these compounds can further enhance their antitumor effects [138,139,140]. The anticancer effects of disulfiram are primarily linked to copper-dependent ROS generation, inhibition of the NF-κB/ubiquitin-proteasome pathway, and modulation of tumor stemness and drug-resistant phenotypes. However, the clinical translation of disulfiram remains constrained by issues related to pharmacokinetic stability and inconsistent clinical benefits [48,68,135].

### 4.4. Repurposing Neuropsychiatric and Other Drugs

Certain drugs originally developed for neuropsychiatric disorders, particularly specific antipsychotics, have demonstrated promising potential for drug repositioning in lung cancer research [141,142]. The phenothiazine drug trifluoperazine has been shown to inhibit DNA repair, delay the clearance of γH2AX following DNA damage, and impede cell cycle checkpoint recovery, thereby enhancing DNA damage-related cell death in a human non-small cell lung cancer model [123,142]. These effects are typically accompanied by elevated levels of reactive oxygen species (ROS), lysosomal dysfunction, vacuolization, and increased apoptosis. Although these ‘serendipitous discoveries’ may not necessarily represent the primary focus for clinical advancement, they provide valuable insights for anticancer drug screening and underscore the broad potential of drug repositioning [73,143].

## 5. Synergistic Mechanisms of Combination Therapy with Natural Products and Marketed Drugs

### 5.1. In Combination with Chemotherapy

The combination of natural products with conventional chemotherapeutic agents represents one of the most frequently explored and translationally promising modalities in the treatment of lung cancer [22,144,145]. The basic principle is not simply to increase toxicity; rather, it first weakens tumor cells to a certain extent through the use of natural products. The details of the combination of these compounds are summarized below (Table 3).

**Figure 2:**
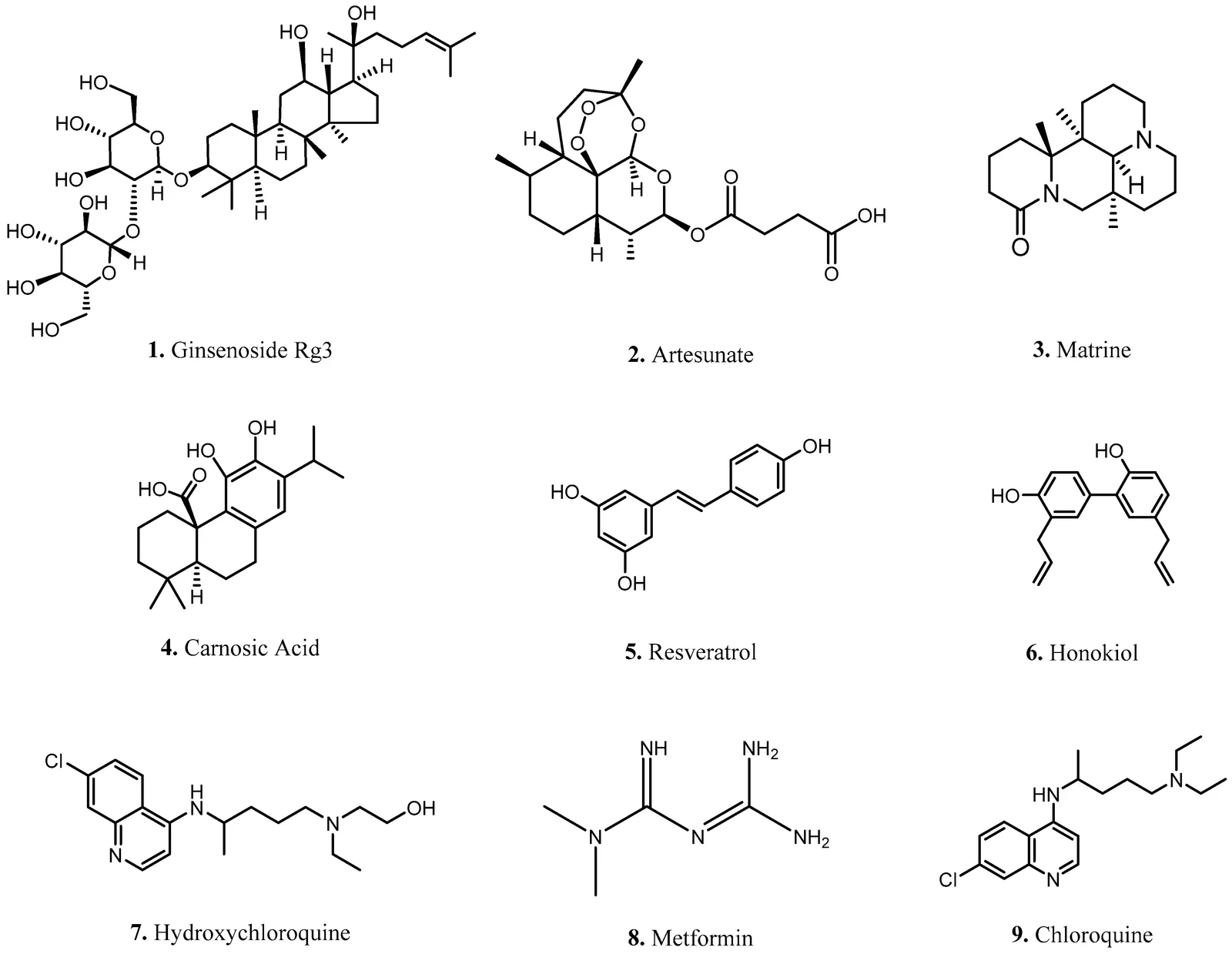
Structure of natural products and marketed drugs used in combination chemotherapy.

Ginsenoside Rg3 (**1**, Figure 2), a natural compound derived from ginseng, exhibits broad-spectrum anticancer activity and is capable of attenuating chemoresistance and radioresistance. Multiple clinical studies have shown that the combination of this compound with chemotherapy can provide multiple benefits in enhancing antitumor efficacy, improving immune function, reducing chemotherapy toxicity, and inhibiting angiogenesis in patients with NSCLC, ultimately achieving improved disease control, prolonged overall survival, and enhanced quality of life [146,147]. It represents a promising adjuvant therapeutic option but still requires further high-quality research for validation [148,149,150]. The natural alkaloid matrine also possesses a wide spectrum of biological activities (**3**, Figure 2). In in vitro cellular experiments, it can inhibit the growth, promote apoptosis, and suppress cell migration of non-small cell lung cancer A549 cells, thereby exerting certain regulatory effects on lung cancer [151]. Multiple clinical studies have compared the efficacy of matrine combined with platinum-based doublet chemotherapy (PBDC) versus PBDC alone in the treatment of advanced non-small cell lung cancer (NSCLC). The results indicate that the combination therapy improved the mean survival time (MST) and quality of life (QOL) while also reducing the incidence of adverse reactions compared with PBDC alone (*p* < 0.05). However, these conclusions need to be verified through strictly controlled randomized trials in the future before definitive conclusions can be drawn regarding this therapy [152,153].

Drug repositioning presents distinct advantages in this context. Metformin has been shown to enhance the sensitivity of tumor cells to chemotherapy through metabolic reprogramming. Additionally, chloroquine and hydroxychloroquine can diminish the buffering and adaptive capacities of tumor cells to chemotherapeutic stress by inhibiting autophagy [149,154]. Evidence from lung cancer studies indicates that autophagy inhibition can amplify the antitumor effects of topotecan and drugs related to the PI3K/mTOR pathway [155,156]. Furthermore, an early clinical study investigating the combination of hydroxychloroquine and erlotinib in advanced non-small cell lung cancer (NSCLC) supports its potential feasibility [157,158,159]. A large number of studies have combined these already marketed drugs with chemotherapy, but the results have not been encouraging. Metformin did not consistently improve PFS/OS in most studies [160,161] (**8**, Figure 2). HCQ/CQ, as autophagy inhibitors, can enhance chemotherapy sensitivity in experiments and a few clinical trials, but clinical outcomes have been inconsistent (**7** and **9**, Figure 2). Overall, the effects of these combination therapies are not stable, and more clinical trial cases are needed for validation and exploration [162,163].

More broadly, certain marketed drugs with potential for repositioning can enhance the effects of chemotherapy by inducing oxidative stress, disrupting lysosomal function, or impairing DNA damage repair mechanisms. However, these effects are highly dependent on the specific drugs, dosages, and tumor backgrounds involved and cannot be generalized as a universal characteristic of all anti-infective agents [11,164]. For patients with NSCLC who exhibit significant drug resistance, these combination regimens still require further biomarker-stratified studies [165,166,167].

**Table 3:** Detailed information on natural products and marketed drugs used in combination chemotherapy.

No	Compound	Sources	Mechanism	Level of Evidence	References
**1**	Ginsenoside Rg3	Panax ginseng	(1) Improvement of antioxidant and anti-inflammation properties; (2) immune regulation; (3) induction of tumor apoptosis; (4) prevention of tumor invasion and metastasis; (5) reduction of chemoresistance and radioresistance	Clinical trial	[148,168]
**2**	Artesunate	Semi-synthetic derivative of artemisinin	(1) Alleviation of the inflammatory response; (2) suppression of fibrosis; (3) promotion of apoptosis	Clinical trial	[48,169,170]
**3**	Matrine	The root bark of Sophora flavescens	(1) Suppression of A549 cell migration; (2) reduction of vascular endothelial growth factor A (VEGF-A) secretion in A549 cells	Clinical trial	[151]
**4**	Carnosic Acid	The rosemary plant	(1) Enhancement of CD8^+^ T cell lethality (suppression of MDSC by carnosic acid)	In vivo	[171]
**5**	Resveratrol	A large variety of plant species	(1) Inhibition of ADAM9 expression	In vitro	[172]
**6**	Honokiol	Magnolia officinalis	(1) Promotion of cell cycle arrest; (2) induction of apoptosis; (3) inhibition of tumor growth; (4) inhibition of angiogenesis, and so on	In vivo	[173]
**7**	Hydroxychloroquine	Synthetic analogs of quinine	(1) Suppression of autophagy	Clinical trial	[162]
**8**	Metformin	Synthetic compounds	(1) AMPK-dependent (direct) anticancer effects; (2) selectively targeting cancer stem cells (CSCs); (3) reversing multidrug resistance (MDR); (4) inhibition of tumor metastasis	Clinical trial	[161]
**9**	Chloroquine	Synthetic analogs of quinine	(1) Suppression of autophagy	Clinical trial	[163]

### 5.2. Combination with Targeted Therapy

**Figure 3:**
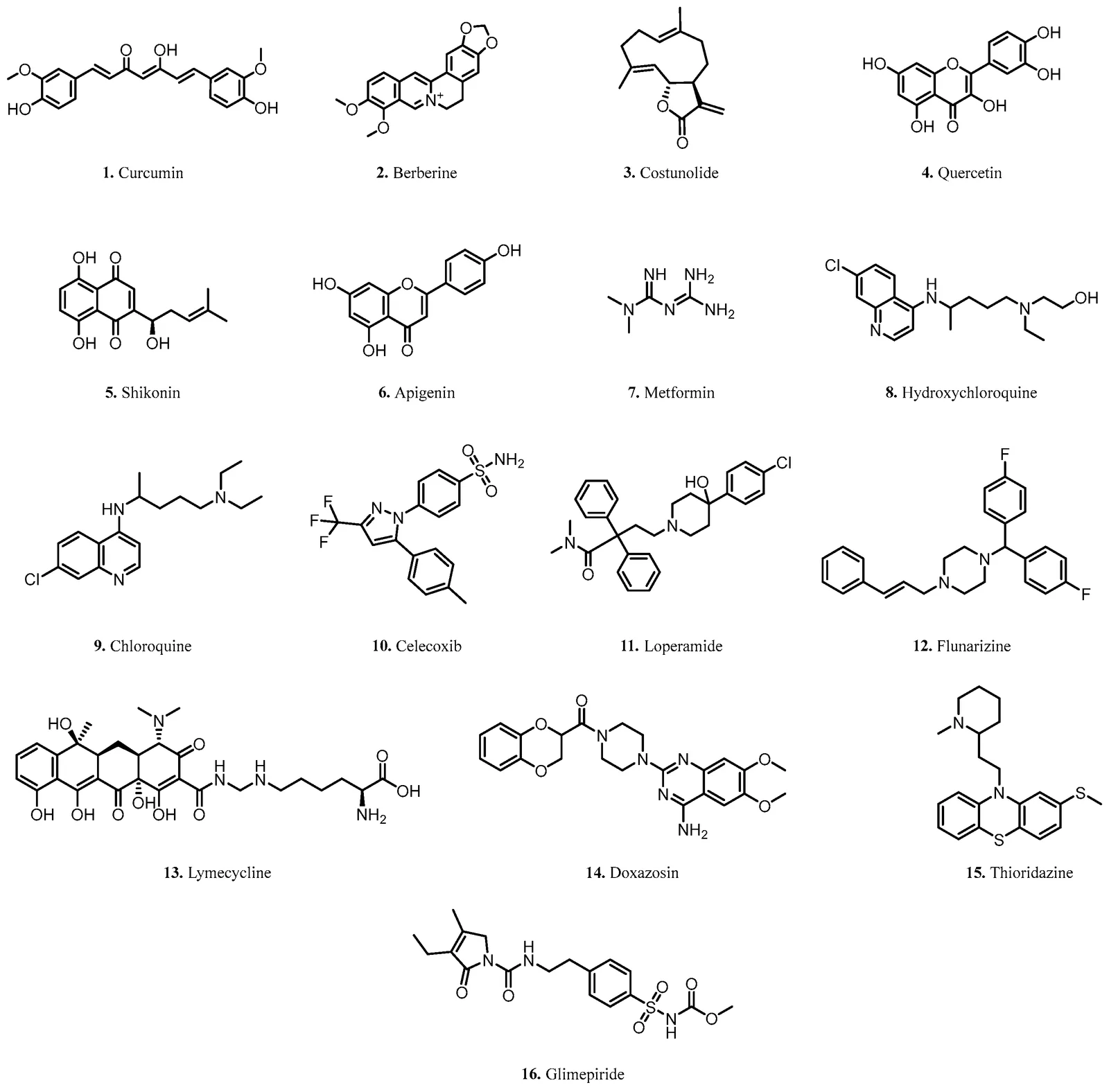
Structure of natural products and marketed drugs used in combination with targeted therapy.

Targeted drugs are antitumor agents designed against specific gene mutations, protein targets, or signaling pathways unique to tumor cells. They can specifically identify and attack cancer cells while causing less damage to normal tissues. Compared with chemotherapy mentioned earlier, they offer advantages such as high selectivity, precise targeting, and fewer side effects. Currently, mainstream targeted drugs for lung cancer mainly act on driver genes such as EGFR, ALK, ROS1, MET, and KRAS. For example, gefitinib (an EGFR-TKI), osimertinib (a third-generation EGFR-TKI), and crizotinib (an ALK-TKI) are all used in the treatment of lung cancer. Due to the inevitable emergence of drug resistance in lung cancer, which poses significant therapeutic challenges, there is an urgent need to explore new treatment strategies to overcome resistance. Natural compounds, with their inherent multi-target capabilities and favorable safety profiles, represent a promising approach to tackling this resistance. Below, we present some details on the application of natural products combined with targeted therapy. Additional details on more compounds are provided in Table 4 (**1–6**, Figure 3).

Curcumin, a turmeric-derived polyphenol, exhibits broad anti-inflammatory and anticancer activities [174] (**1**, Figure 3). In vivo and in vitro, it reverses cisplatin resistance in lung cancer cells [174]. Combined with gefitinib, curcumin disrupts Sp1-HDAC1 interaction to downregulate EGFR and suppress ERK/MEK and AKT/S6K signaling in resistant NSCLC, triggering autophagy-dependent cell death; thus, it may sensitize EGFR-TKIs in EGFR-wild-type or KRAS-mutant NSCLC [175]. Similarly, curcumin plus erlotinib enhances apoptosis; reduces EGFR, p-EGFR and Survivin; inhibits NF-κB; and suppresses in vivo tumor growth in erlotinib-resistant NSCLC, supporting curcumin as a promising adjuvant [176,177]. Berberine, an isoquinoline alkaloid from traditional Chinese herbs, possesses cardioprotective and broad-spectrum anticancer activities against various malignancies [178] (**2**, Figure 3). In vivo and in vitro, it suppresses lung cancer progression by inducing apoptosis, autophagy and mitochondrial dysfunction while inhibiting cell proliferation, cycle progression and migration [179,180]. Notably, berberine reverses the epithelial–mesenchymal transition (EMT) in osimertinib-resistant NSCLC cells via upregulating epithelial markers and downregulating mesenchymal markers. It also blocks the Notch1 signaling pathway in resistant cells, further overcoming osimertinib resistance [180,181]. Collectively, berberine synergizes with osimertinib to reverse NSCLC drug resistance, providing a solid theoretical foundation for subsequent clinical translation [180,182].

To date, there have been numerous studies investigating the combination of already marketed drugs with targeted therapy for the treatment of lung cancer. Drug repositioning offers a new research direction for overcoming resistance to targeted therapy in lung cancer [183,184,185]. Various older marketed drugs have been found to exhibit synergistic effects when combined with targeted agents such as EGFR-TKIs, particularly in EGFR-TKI-resistant NSCLC. Metformin is a representative metabolic repositioning drug that enhances the anti-lung cancer activity of gefitinib, erlotinib, or osimertinib by activating AMPK, inhibiting mTOR/IGF-1R-related signaling, and regulating processes such as IL-6/STAT3 and EMT in vivo and in vitro [186] (**7**, Figure 3). However, two randomized clinical trials have failed to demonstrate stable therapeutic benefits of metformin combined with targeted therapy, suggesting that further rigorous investigations are still warranted [187,188]. Old antimalarial/antirheumatic drugs represented by Hydroxychloroquine and Chloroquine mainly delay or reverse drug resistance by blocking the autophagy–lysosome pathway, attenuating EGFR-TKI-induced protective autophagy and drug-tolerant persister formation (**8** and **9**, Figure 3). Likewise, a randomized clinical trial yielded negative results for a hydroxychloroquine-targeted-therapy combination, with no apparent therapeutic effect observed [157,189]. In addition, celecoxib can enhance the inhibitory effect of gefitinib on NSCLC cells by inhibiting the COX-2/PGE2 axis and enhancing blockade of pathways such as AKT, ERK, and NF-κB [157] (**10**, Figure 3). Nevertheless, celecoxib likewise failed to elicit obvious therapeutic effects in clinical trials. More research details are described in Table 4. These studies suggest that old drugs already on the market can provide candidate strategies for sensitizing targeted therapy and reversing drug resistance in lung cancer by modulating multiple mechanisms, including metabolism, inflammation, autophagy, epigenetics, EMT, and bypass survival signaling [143,190]. It is foreseeable that more repurposed old drugs will be investigated for combination with targeted therapies in lung cancer treatment in future studies [183,191,192,193].

Overall, at the molecular level, interfering with lung cancer progression and drug resistance through multiple signaling pathways has become a key entry point for research on the repurposing of natural products and marketed drugs [118,143,194]. It is important to emphasize that the core value of such strategies does not lie in the complete replacement of targeted drugs but rather in delaying the evolution of resistance, improving the likelihood of sustained remission, and enhancing long-term efficacy through multipathway synergy [143,154,195]; however, at this stage, the evidence primarily derives from cellular and animal models, necessitating further validation through biomarker stratification, re-biopsy for drug resistance, and prospective combination trials [185,196,197].

**Table 4:** Detailed information on natural products and marketed drugs used in targeted therapy.

No	Compound	Sources	Mechanism	Combined Targeted Therapy	Level of Evidence	References
**1**	Curcumin	Herbal remedy and dietary spice turmeric	(1) Inhibition of EGFR activity and its downstream MEK/ERK and AKT/S6K pathways (Gefitinib) (2) Downregulation of EGFR, p-EGFR and survivin; inhibition of NF-κB activation; and enhancement of erlotinib-induced apoptosis (Erlotinib)	Gefitinib; Erlotinib	In vivo	[174,175,176,177,198,199]
**2**	Berberine	Medicinal plants such as Hydrastis canadensis, Berberis aristata, Coptis chinensis	(1) Inhibition of MET kinase activity and downstream PI3K/AKT/mTOR and MEK/ERK signaling (2) Upregulation of Bim, downregulation of Mcl-1, and promotion of apoptosis (3) Inhibition of Notch1 signaling, reversal of EMT, and restoration of sensitivity to osimertinib	Osimertinib	In vivo	[178,179,180]
**3**	Costunolide	Costus (Saussurea lappa Clarke) root	(1) Dual inhibition of MEK1 and AKT1/2 (2) Blockade of ERK/RSK and AKT/GSK3β/NF-κB signaling	Osimertinib	In vivo	[200,201,202]
**4**	Quercetin	From quercetum (oak forest)	(1) Direct binding and inhibition of G6PD (2) Reduction of NADPH levels (3) Promotion of EGFR T790M protein degradation (4) Consequent enhancement of gefitinib sensitivity	Gefitinib	In vivo	[203,204]
**5**	Shikonin	Lithospermum erythrorhizon	(1) Modulation of tumor energy metabolism, reduction of OCR and GlycoPER; downregulation of PKM2, p-EGFR, P-gp and HIF-1α, and increased intracellular accumulation of gefitinib	Gefitinib	In vivo	[205,206]
**6**	Apigenin	Apium genus such as Chinese celery and parsley	(1) Inhibition of HIF-1α, c-Myc and p-EGFR (2) Reduction of GLUTs and MCT1 to disrupt glucose metabolism; blockade of autophagic flux and induction of apoptosis	Gefitinib	In vitro	[207,208]
**7**	Metformin	Synthetic compound	(1) Activation of AMPK and suppression of mTOR/IGF-1R-associated metabolic and growth signaling (2) Inhibition of the IL-6/STAT3 axis, reversion of EMT (3) Enhancement of EGFR-TKI-induced apoptosis	Gefitinib/Erlotinib/Icotinib/Osimertinib	Clinical trial	[186]
**8**	Hydroxychloroquine	Synthetic analogs of quinine	(1) Inhibition of lysosomal acidification and autophagic flux (2) Attenuation of protective autophagy in tumor cells under EGFR-TKI stress	Erlotinib/Gefitinib	Clinical trial	[157]
**9**	Chloroquine				In vivo	
**10**	Celecoxib	Synthetic compound	Inhibition of AKT and ERK pathways in NSCLC, as well as suppression of EGFR expression upon high-concentration treatment	Gefitinib	Clinical trial	[209,210,211]
**11**	Loperamide	Synthetic compound	Enhancement of apoptosis and G1-phase cell cycle arrest	Gefitinib	In vitro	[212]
**12**	Flunarizine	Synthetic compound	Upregulation of the pro-apoptotic protein Bim and the cell cycle inhibitor p27Kip1, and downregulation of Bcl-2	Gefitinib	In vivo	[213]
**13**	Lymecycline	Synthetic compound	(1) Inhibition of EGFR phosphorylation and its downstream AKT/ERK/STAT3 signaling (2) Induction of cell cycle arrest and apoptosis	Icotinib	In vivo	[214]
**14**	Doxazosin	Synthetic compound	(1) Induction of cytotoxic autophagy	Osimertinib	In vitro	[215]
**15**	Thioridazine	Synthetic compound	(1) Reduction of p-AKT levels and promotion of apoptosis	Gefitinib	In vitro	[216]
**16**	Glimepiride	Synthetic compound	(1) Activation of AMPK followed by suppression of ERK/MMP7 signaling	EGFR-TKIs	In vivo	[217]

### 5.3. Combined with Radiotherapy

Radiotherapy primarily relies on ionizing radiation to induce DNA double-strand breaks, which are accompanied by the accumulation of reactive oxygen species (ROS), thereby triggering tumor cell death. However, tumor cells often develop radioresistance through mechanisms such as the Nrf2-driven antioxidant network, the GSH-GPX4 axis, and enhanced DNA damage repair capacity [218,219,220,221]. Therefore, natural products and existing drugs that can amplify oxidative stress, deplete GSH, inhibit Nrf2 signaling, or interfere with DNA repair present a theoretical basis for their use as radiosensitizers [220,221,222].

**Figure 4:**
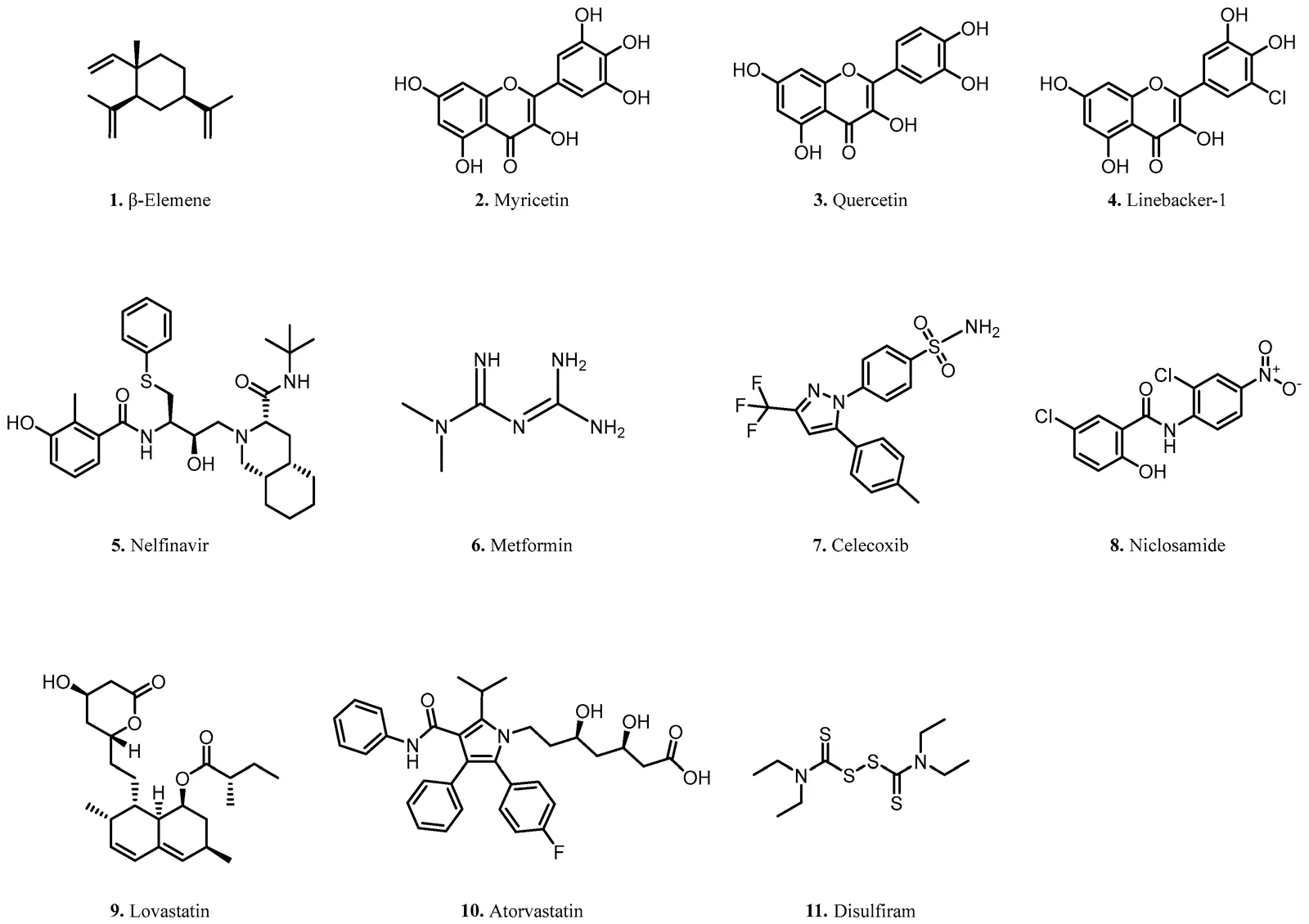
Structure of natural products and marketed drugs used in combination with radiotherapy.

In recent years, a variety of natural products and their derivative preparations have been explored for strategies to enhance radiosensitivity in lung cancer radiotherapy. β-Elemene can increase the sensitivity of NSCLC to ionizing radiation/X-ray irradiation by inhibiting EMT, cancer stemness, and the Prx-1/NF-κB/iNOS signaling axis, as well as enhancing DNA damage and inhibiting Rad51-associated repair [223] (**1**, Figure 4). Myricetin and quercetin, as flavonoids, enhance the sensitivity of lung cancer cells to X-ray irradiation by promoting Caspase-3-related apoptosis and regulating the miR-16-5p/WEE1 axis, respectively, in vivo and in vitro [204,224,225,226,227] (**2** and **3**, Figure 4). Details are provided in Table 5.

Drug repositioning has provided new research directions for radiosensitization in lung cancer [22,228,229]. Multiple already-marketed old drugs have been reassessed as radiation sensitizers, including the anti-HIV drug nelfinavir (**5**, Figure 4), the antidiabetic drug metformin (**6**, Figure 4), the COX-2 inhibitor celecoxib (7, Figure 4), the anthelmintic drug niclosamide (**8**, Figure 4), the lipid-lowering drugs statins, the autophagy inhibitors chloroquine/hydroxychloroquine, and the anti-alcoholism drug disulfiram (**11**, Figure 4), among others. Nelfinavir is a case with relatively sufficient clinical translational evidence. Its combination with external-beam thoracic radiotherapy or concurrent chemoradiotherapy for unresectable locally advanced NSCLC may enhance the effect of radiotherapy by inhibiting PI3K/AKT, downregulating HIF-1α/VEGF, and improving tumor oxygenation [230]. Metformin, as a metabolic old drug, has also been studied in concurrent chemoradiotherapy for locally advanced NSCLC (**6**, Figure 4). However, the randomized phase II OCOG-ALMERA trial did not show clear benefit, suggesting that its clinical application still requires further biomarker screening and population optimization [231,232]. At the preclinical level, niclosamide can reverse acquired radioresistance in lung cancer by blocking the JAK2/STAT3/Bcl-2/Bcl-XL pathway [233,234] (**8**, Figure 4); celecoxib can enhance the killing effect of X-ray irradiation by inhibiting COX-2/PGE2 and EGFR nuclear translocation/DNA-PK-mediated DNA repair [235,236] (**7**, Figure 4); statins such as lovastatin and atorvastatin increase the sensitivity of lung cancer cells to ionizing radiation by affecting the mevalonate pathway, AKT/AMPK, ROS, and apoptosis [237,238,239,240,241] (**9** and **10**, Figure 4). Similarly, celecoxib still failed to yield significant clinical improvements when combined with concurrent chemoradiotherapy in clinical trials [242]. These studies suggest that already-marketed old drugs can enhance the efficacy of radiotherapy for lung cancer through mechanisms such as metabolic regulation, DNA repair inhibition, hypoxia improvement, autophagy blockade, and immune microenvironment remodeling. However, except for a few drugs that have undergone early clinical studies, most are still in the preclinical stage [219,221,222]. In addition to monomeric compounds, there are also compound formulations. For example, S-1 (Tegafur/Gimeracil/Oteracil) is an oral anticancer compound formulation composed of three synthetic small molecules precisely proportioned, which also exerts anticancer effects when used in combination with radiotherapy [243].

**Table 5:** Detailed information on natural products and marketed drugs used in radiotherapy.

No	Compound	Sources	Mechanism	Combined Radiotherapy	Level of Evidence	References
**1**	β-Elemene	Curcuma wenyujin	(1) Inhibition of EMT and CSC phenotypes (2) Suppression of the Prx-1/NF-κB/iNOS signaling axis	Ionizing radiation/X-ray irradiation	Clinical trial	[223]
**2**	Myricetin	Many natural plants	(1) Upregulation of Caspase-3 (2) Promotion of radiation-induced apoptosis	X-ray irradiation	In vivo	[224,225]
**3**	Quercetin	Fruits and vegetables	(1) Upregulation of miR-16-5p, inhibition of WEE1, disruption of the G2/M checkpoint and suppression of radioresistance	X-ray irradiation	In vitro	[204,226]
**4**	Linebacker-1	NP-derived semisynthetic drug	(1) Promotion of radiation-induced apoptosis	IGRT/X-ray irradiation	In vivo	[227]
**5**	Nelfinavir	Synthetic compound	(1) Inhibition of the PI3K/AKT survival pathway (2) Downregulation of HIF-1α/VEGF, improvement of tumor oxygenation, and reduction of hypoxia-associated radioresistance	External-beam thoracic radiotherapy; 3D conformal radiotherapy, 3D-CRT; intensity-modulated radiotherapy, IMRT	Clinical trial	[230,244]
**6**	Metformin	Synthetic compound	(1) Activation of AMPK, inhibition of mTOR and suppression of tumor metabolic adaptation	Concurrent external-beam thoracic radiotherapy/concurrent chemoradiotherapy	Clinical trial	[231,232]
**7**	Celecoxib	Synthetic compound	(1) Inhibition of the pro-inflammatory and pro-survival COX-2/PGE2 axis; (2) Enhancement of apoptosis and cell cycle arrest	X-ray irradiation/ionizing radiation	Clinical trial	[235,236]
**8**	Niclosamide	Synthetic compound	(1) Inhibition of the JAK2/STAT3/Bcl-2/Bcl-XL survival pathway and reduction of STAT3 nuclear localization (2) Blockade of anti-apoptotic signals and restoration of radiotherapy-induced cell death	Ionizing radiation/X-ray irradiation	In vivo	[233,234]
**9**	Lovastatin	Aspergillus terreus	(1) Reduction of EGFR/AKT-associated survival signals; concurrent enhancement of AMPK phosphorylation and induction of apoptosis	Ionizing radiation	In vitro	[237,238]
**10**	Atorvastatin	Synthetic compound	(1) Augmentation of ROS production, promotion of apoptosis and radiation-induced cell death	Ionizing radiation	In vitro	[239]
**11**	Disulfiram	Synthetic compound	(1) Potential regulation of radiosensitivity via the NF-κB pathway (2) Concurrent upregulation of PD-L1 and remodeling of the post-radiation tumor immune microenvironment	Ionizing radiation	In vivo	[245]

### 5.4. Combination with Immunotherapy

Immune checkpoint inhibitors have emerged as a crucial cornerstone in the treatment of advanced non-small cell lung cancer (NSCLC) [109,122,246,247]. However, their overall response rate remains limited, and their efficacy is significantly influenced by the tumor immune microenvironment, PD-L1 expression status, and antigen presentation capacity [248,249,250]. Consequently, natural products and drug repositioning molecules that can remodel the immune microenvironment are increasingly recognized as promising candidates for combination immunotherapy [28,251,252,253,254,255,256].

**Figure 5:**
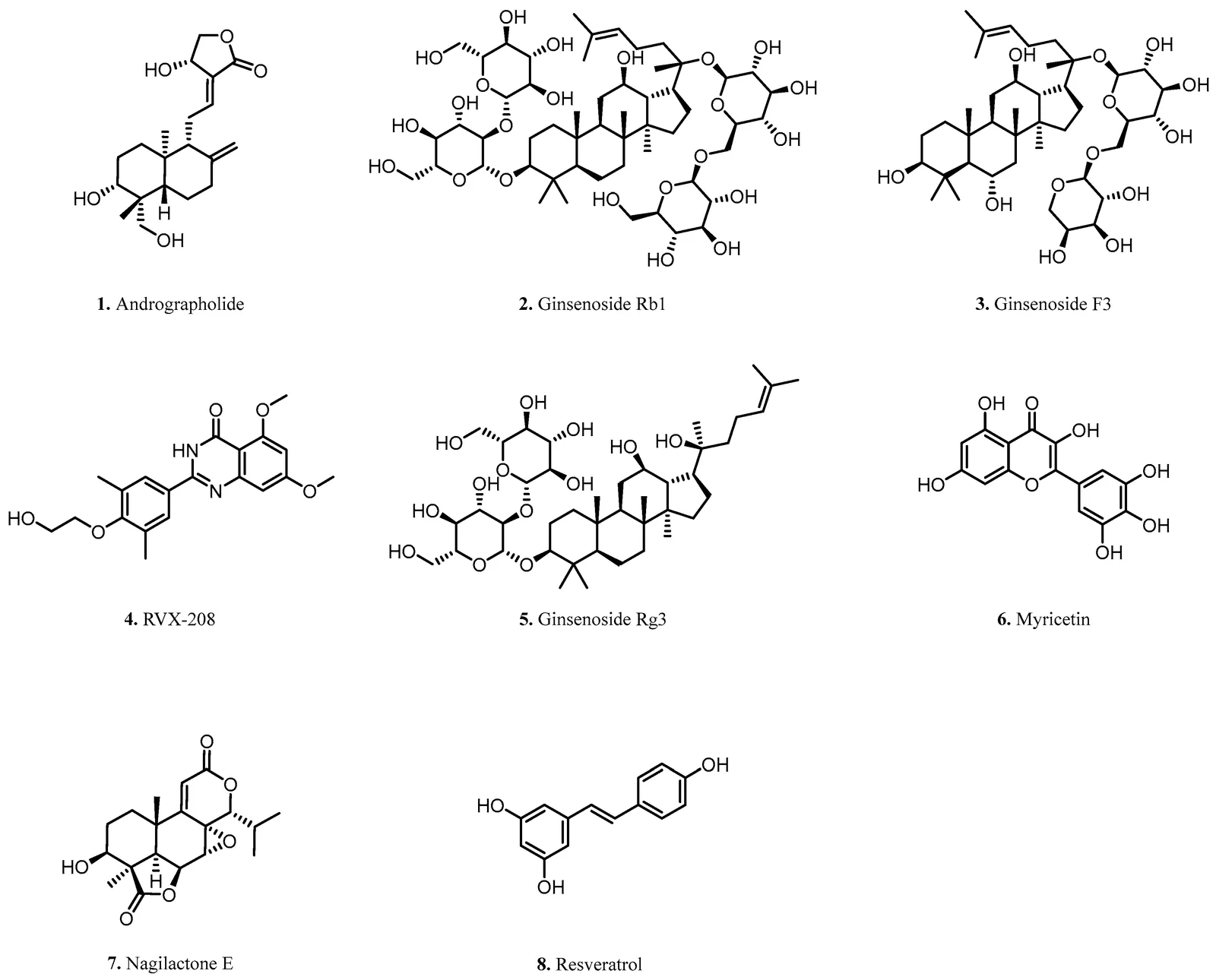
Structure of natural products and marketed drugs used in combination with immunotherapy.

In recent years, natural products have been widely used to explore strategies for sensitizing lung cancer immunotherapy, with a particular focus on enhancing the efficacy of immunotherapy and reversing immunotherapy resistance [251,253,254,257]. Ginsenosides are representative natural products [258,259], among which Ginsenoside Rb1 (**2**, Figure 5), when delivered via TMTP1 peptide-enhanced exosomes, can enhance the efficacy of nivolumab in a PI3K-mutated NSCLC model of acquired immunotherapy resistance in vivo. The underlying mechanism involves inhibition of the PI3K/AKT/mTOR pathway, promotion of M1 macrophage polarization, and enhancement of CD8^+^ T cell proliferation and cytotoxicity [260,261,262]. Ginsenoside F3 alleviates T-cell exhaustion via RIPOR2-mediated immunometabolic reprogramming, enhancing anti-PD-1 therapy in a mouse model of NSCLC in vivo [263] (**3**, Figure 5). In addition to ginsenosides, andrographolide can enhance anti-PD-1 therapy by suppressing the COX2/PGE2 immunosuppressive axis and promoting CD8+ T-cell infiltration and functional recovery in vivo [264,265,266] (**1**, Figure 5). Furthermore, resveratrol-related nanoformulations and derivatives can enhance the immunotherapy response in lung cancer by improving CD8+ T-cell metabolism or inhibiting the JAK2-PD-1/PD-L1 axis in vivo and in vitro [267,268] (**12,** Figure 5). Please refer to Table 6 for more details.

The combination of marketed drugs and immune checkpoint inhibitors provides an important strategy for drug repurposing and rapid translation in lung cancer treatment [249,251,252,269,270]. Current studies have shown that marketed single drugs capable of synergizing with immunotherapy are primarily concentrated in categories such as anti-angiogenic drugs, DNA damage repair inhibitors, transcription-inhibitory cytotoxic drugs, and inflammatory pathway modulators. The phase III CANOPY-1 trial enrolled 643 patients with advanced/metastatic NSCLC without EGFR or ALK alterations. The median PFS was 6.8 months in both the canakinumab and placebo groups (HR = 0.85; *p* = 0.102). Median OS was 20.8 versus 20.2 months, respectively (HR = 0.87; *p* = 0.123). Neither difference reached statistical significance. Accordingly, the addition of the IL-1β inhibitor canakinumab failed to improve survival outcomes of first-line chemoimmunotherapy [271]. Therefore, the combination of marketed single drugs with immunotherapy represents an important direction for optimizing lung cancer treatment. However, its success depends on a clear mechanistic basis, appropriate disease context, and biomarker-guided patient selection.

**Table 6:** Detailed information on natural products and marketed drugs used in immunotherapy.

No	Compound	Sources	Mechanism	Combined Immunotherapy	Level of Evidence	References
**1**	Andrographolide	Andrographis paniculata	(1) Inhibition of COX2 activity and PGE2 release, alleviation of PGE2-mediated immunosuppression	Anti-PD-1 antibody	In vivo	[264,265]
**2**	Ginsenoside Rb1	Panax ginseng	(1) Inhibition of the PI3K/AKT/mTOR pathway (2) Promotion of M1 macrophage polarization	Nivolumab/anti-PD-1 therapy	In vivo	[260,261,262]
**3**	Ginsenoside F3	Panax ginseng	(1) Restoration of RIPOR2-mediated immunometabolic regulation for alleviation of T cell exhaustion	Anti-PD-1 therapy	In vivo	[263]
**4**	RVX-208	Resveratrol derivative	(1) Interference with JAK2 phosphorylation (2) Reduction of PD-1 expression in T lymphocytes and PD-L1 expression in lung cancer cells	Anti-PD-1 antibody	In vitro	[268]
**5**	Ginsenoside Rg3	Panax ginseng	(1) Reduce PD-L1 glycosylation to reduce steric hindrance and enhance antigen-binding ability (2) Promotion of PD-L1 destabilization or reduction of functional PD-L1, thereby restoration of T cell-mediated cytotoxicity	Atezolizumab	In vivo	[258,259,272]
**6**	Myricetin	Many plants	(1) Inhibition of the JAK-STAT-IRF1 pathway and reduction of PD-L1 and IDO1 expression	PD-1/PD-L1 blockade	In vitro	[273,274]
**7**	Nagilactone E	Podocarpus nagi	(1) Upregulation of PD-L1 via the JNK-c-Jun axis, enhancement of tumor reliance on the PD-1/PD-L1 immune evasion pathway	PD-L1 inhibitors	In vitro	[275]
**8**	Resveratrol	Many plants	(1) Targeting of CD93 for modulation of the CD93–AKT–PAK5–AIF axis (2) Promotion of AIF mitochondrial translocation	Anti-PD-1 therapy	In vivo	[267]
**9**	Canakinumab	Recombinant humanized monoclonal antibody	(1) High-affinity anti-IL-1β monoclonal antibody canakinumab	Pembrolizumab	Clinical trial	[271,276]

### 5.5. Summary of Shared Targeting Mechanisms

**Figure 6:**
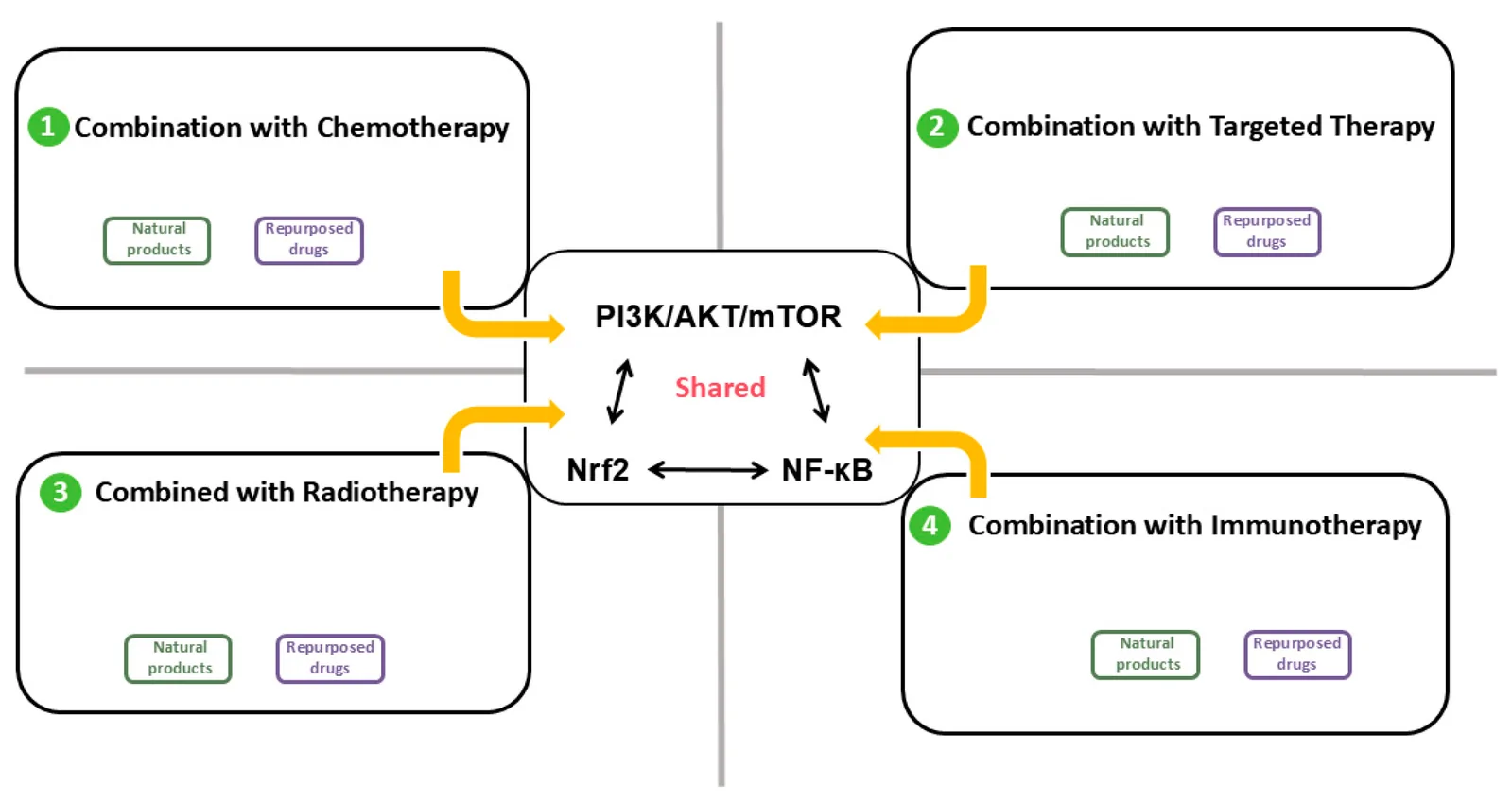
Core molecular pathways co-regulated across the four combination therapy modalities.

The preceding sections have summarized the mechanisms of natural products and repurposed drugs in NSCLC for four combination-therapy settings: chemotherapy, targeted therapy, radiotherapy, and immunotherapy. Cross-comparison of these findings reveals that despite their diverse origins, these compounds converge on a limited set of core signaling nodes, most of which are supported by documented evidence from at least three of the four combination modalities (Figure 6).

The PI3K/AKT/mTOR axis is the most frequently explicitly mentioned node among targeted-therapy, radiotherapy and immunotherapy combination settings. Multiple descriptions of its inhibition appear in targeted-therapy entries, including “inhibition of MET-downstream PI3K/AKT/mTOR and MEK/ERK signaling”, “reduction of p-AKT levels”, “dual inhibition of AKT1/2”, “blockade of EGFR phosphorylation and its downstream AKT/ERK/STAT3 signaling”, and “inhibition of the AKT and ERK pathways upon high-concentration treatment”. For radiotherapy, direct records include “inhibition of the PI3K/AKT survival pathway”, “reduction of EGFR/AKT-associated pro-survival signals”, and “AMPK activation accompanied by mTOR inhibition”. For immunotherapy, “inhibition of the PI3K/AKT/mTOR pathway” is explicitly noted. Although PI3K, AKT or mTOR are not explicitly mentioned under chemotherapy-combination entries, phenotypes such as “reversal of multidrug resistance”, “inhibition of angiogenesis”, and “AMPK-dependent effects” are highly consistent with this pathway. These observations suggest that the PI3K/AKT/mTOR axis acts as a well-defined convergent node across the latter three combination modalities, whereas in chemotherapy-based combinations, it functions largely through parallel mechanisms involving AMPK and apoptosis.

NF-κB functions as a shared inflammation–pro-survival axis across targeted-therapy, radiotherapy, and immunotherapy combination regimens. Two explicit entries are documented for targeted-therapy combinations: “inhibition of NF-κB activation (erlotinib entry)” and “blockade of AKT/GSK3β/NF-κB signaling”. For radiotherapy, direct evidence includes “inhibition of the Prx-1/NF-κB/iNOS axis” and “suppression of the pro-inflammatory and pro-survival COX-2/PGE_2_ axis” (COX-2 is a canonical target gene of NF-κB). In the immunotherapy combination section, “suppressed release of COX-2/PGE_2_” is also linked to NF-κB activity. Although NF-κB is not explicitly mentioned in chemotherapy-combination entries, observations such as “improved anti-inflammatory properties”, “carnosic acid-mediated suppression of MDSCs to potentiate CD8^+^ T-cell cytotoxicity”, and “alleviation of inflammatory responses” correspond to NF-κB-controlled inflammatory-cytokine and myeloid-derived suppressor-cell programs, which can be considered phenotypic readouts of this axis in chemotherapy-based combinations.

Nrf2 was only briefly mentioned in the original manuscript under general descriptions of “antioxidant activity/oxidative stress”. Nevertheless, item-by-item inspection reveals traces of Nrf2 involvement across all four combination regimens. In chemotherapy combinations, “improved antioxidant and anti-inflammatory properties” and “reduced chemoradiotherapy resistance” directly correspond to the HO-1/NQO1/GCLC transcriptional program mediated by this axis. In targeted-therapy combinations, “direct binding-mediated inhibition of G6PD accompanied by NADPH reduction”, “downregulation of HIF-1α and c-Myc”, as well as “AMPK activation/mTOR inhibition”, may all relate to upstream regulation of Nrf2 or the expression of its downstream target genes. In radiotherapy combinations, the observed “enhanced ROS production” and “disturbed AMPK/mTOR-driven metabolic adaptation” indicate perturbation of tumor cell redox homeostasis, which may crosstalk with Nrf2-associated redox signaling. For immunotherapy combinations, “inhibition of PI3K/AKT/mTOR” and “reduced OCR/GlycoPER” also exhibit crosstalk with this redox–regulatory axis.

Taken together, both natural products and repurposed drugs inevitably exert complex regulatory mechanisms in vivo. Most of these mechanisms are not independent, parallel events but are highly intertwined with upstream-and-downstream relationships. Nevertheless, natural products and repurposed drugs described in this chapter converge on core molecular hubs, including PI3K/AKT/mTOR, NF-κB and Nrf2, across the four NSCLC combination-therapy settings, forming a unified mechanistic framework shared by diverse combinatorial strategies.

### 5.6. Context-Dependent Effects of Metformin, Chloroquine and Hydroxychloroquine Under Distinct Combination Therapy Modalities

In Chapter 5, several compounds exhibit similar molecular mechanisms across diverse combination settings, yet their functional significance varies substantially under different therapeutic modalities. To illustrate this context-dependent feature, this section focuses on metformin, chloroquine (CQ), and hydroxychloroquine (HCQ). Metformin primarily modulates mitochondrial energy metabolism and the AMPK/mTOR signaling axis, whereas CQ/HCQ mainly disrupts lysosomal function and autophagic flux. Importantly, their biological roles differ considerably when combined with chemotherapy, targeted therapy, or radiotherapy. Such discrepancies arise from distinct therapeutic stresses imposed by each treatment modality: chemotherapy induces acute cytotoxicity and DNA damage, targeted therapy blocks oncogenic driver signaling, and radiotherapy mainly triggers oxidative stress, DNA double-strand breaks, and hypoxia-associated radioresistance. Accordingly, the synergistic effects of these repurposed drugs cannot be simply summarized as “AMPK activation” or “autophagy inhibition”. Instead, their therapeutic values consist in the targeted interruption of adaptive survival mechanisms employed by NSCLC cells under diverse treatment stresses.

#### 5.6.1. Metformin

(1) Combination with Chemotherapy

Chemotherapeutic agents, particularly platinum-based drugs, exert antitumor effects by triggering DNA damage, oxidative stress and apoptosis. Nevertheless, a subset of surviving tumor cells can persist and develop drug resistance through augmented energy metabolism, elevated antioxidant capacity, and enhanced DNA-damage repair, as well as EMT- and stemness-associated phenotypes. Under this scenario, metformin mainly constrains energy supply and anabolic recovery in tumor cells, impairs their repair and repopulation capacity following chemotherapy-inflicted injury, and consequently sensitizes tumor cells to cytotoxic agents [277,278].

(2) Combination with Targeted Therapy

Molecular-targeted agents, represented by EGFR-TKIs, block key oncogenic-driver signals. Even so, tumor cells may sustain survival via activation of bypass signaling such as PI3K/AKT/mTOR, metabolic reprogramming, EMT, or entry into low-proliferative and drug-tolerant states. When combined with targeted therapy, metformin not only augments short-term cytotoxicity but, more importantly, elevates cellular energy stress and constrains metabolic plasticity and compensatory survival capacity of tumor cells [278,279,280]. Accordingly, metformin may help diminish residual tumor-cell populations after targeted therapy and interfere with the evolutionary process of drug resistance.

(3) Combination with Radiotherapy

Radiotherapy exerts cytotoxic effects mainly by generating ROS and triggering DNA damage, whereas tumor hypoxia, maintenance of redox homeostasis, mitochondrial functional adaptation, and DNA-damage repair all contribute to radioresistance. By inhibiting oxidative phosphorylation and reducing oxygen consumption in tumor cells, metformin restricts energy recovery following radiation-induced injury. Under certain circumstances, its oxygen-sparing effect may also ameliorate regional tumor hypoxia [281,282]. Therefore, the potential benefit of combining metformin with radiotherapy primarily lies in impairing the adaptive capacity of tumor cells against radiation-provoked oxidative damage and energetic stress.

#### 5.6.2. Chloroquine and Hydroxychloroquine

(1) Combination with Chemotherapy

Chemotherapy triggers DNA damage, mitochondrial injury and proteostasis imbalance, thereby inducing autophagy in tumor cells. When such autophagy exerts cytoprotective effects, tumor-cell death can be evaded through clearance of damaged mitochondria and other organelles, recycling metabolic substrates, and maintenance of redox homeostasis. CQ/HCQ blocks this self-rescue process, leading to sustained accumulation of damaged organelles, ROS and metabolic stress, and consequently promotes the conversion of chemotherapy-inflicted injury into apoptosis or other forms of irreversible cell death [283,284].

(2) Combination with Targeted Therapy

Following targeted-therapy exposure, subsets of NSCLC cells can enter low-proliferative, dormancy-like or drug-tolerant persister states. These cells are generally insensitive to conventional cytotoxic insults yet may rely on autophagy to sustain mitochondrial quality control, nutrient recycling, and redox balance. Under this context, CQ/HCQ potentially blocks TKI-triggered cytoprotective autophagy and disrupts metabolic homeostasis, as well as the stress-tolerance capacity of residual tumor cells [285,286,287]. Accordingly, the rationale for combining CQ/HCQ with targeted therapy lies more in suppressing residual-cell survival and delaying the expansion of drug-resistant clones rather than merely amplifying the initial therapeutic response [288].

(3) Combination with Radiotherapy

Ionizing radiation induces ROS accumulation, DNA double-strand breaks, mitochondrial damage and endoplasmic reticulum stress. Autophagy may help tumor cells eliminate damaged structures, sustain energy supply and restore cellular homeostasis during this process, thereby serving as a potential radioprotective mechanism. By interfering with the autophagy–lysosomal pathway, CQ/HCQ impedes the clearance of radiation-damaged organelles and further aggravates oxidative stress, mitochondrial dysfunction and the overall burden of cellular injury, thus conferring potential radiosensitizing effects [288,289,290,291].

In summary, even for a given drug with identical molecular-level mechanisms, its biological significance may differ substantially across distinct therapeutic contexts and applications. This subsection aims to dissect these three widely investigated compounds and provide insights for future research.

## 6. Delivery Systems and Formulation Optimization

Although natural products and some marketed drugs exhibit antitumor potential in lung cancer models, their clinical translation is frequently hampered by pharmaceutical obstacles, including poor water solubility, insufficient in vivo exposure, non-targeted distribution, and rapid metabolic clearance. Nanodelivery systems, such as liposomes, polymeric nanoparticles, polymeric micelles, exosomes/extracellular vesicles (EVs), stimuli-responsive carriers, and multidrug co-delivery platforms, can improve the translational prospect of candidate agents by enhancing solubility, stability, cellular uptake and local tissue exposure. Nevertheless, such benefits should not be presumed in general terms; instead, they need to be validated through parameters such as particle size, size distribution, polydispersity index (PDI), surface zeta potential, drug-loading capacity, encapsulation efficiency, release profile, and in vivo pharmacokinetics. The US FDA also mandates systematic characterization of particle-size distribution, zeta potential, encapsulation/drug-loading efficiency, free-drug fraction, in vitro release, drug leakage and stability for liposomal pharmaceutical products, indicating that these metrics are directly linked to product quality and clinical comparability [292].

Pulmonary local drug delivery offers a targeted strategy to overcome insufficient systemic exposure. Taking curcumin as an example, its aqueous solubility is approximately 11 ng/mL at 25 °C, and it suffers from rapid metabolism and insufficient cellular uptake [293,294]. Adel et al. prepared spray-dried curcumin proliposome dry powders. The optimized formulation exhibited a reconstituted particle size of 126.40 ± 3.25 nm, PDI of 0.40 ± 0.01, zeta potential of +31.90 ± 1.69 mV, and encapsulation efficiency of 91.60 ± 0.99%. In rat pulmonary pharmacokinetic studies, this formulation increased the absorption rate, absorption extent and mean residence time in lung tissue relative to free curcumin powder, suggesting that proliposome dry powders could achieve preferential pulmonary exposure [295]. Likewise, quercetin-loaded solid lipid microparticles had a drug-loading capacity of 15.5 ± 0.6%, an encapsulation efficiency of 71.4%, and a median particle size of 2.90 ± 0.30 μm; the fraction of fine particles with an aerodynamic diameter below 4.46 μm was 20.5 ± 3.3%. In the Calu-3 pulmonary epithelial model, 22.32 ± 1.51% transepithelial transport was observed within 4 h, whereas no transepithelial transport was detectable for unformulated quercetin under identical conditions [295,296]. These results indicate that the practical value of lipid-based carriers consists not only in elevating apparent solubility but also in achieving local delivery through inhalable particle size, adequate drug-loading efficiency and improved transepithelial transport. By the same token, micelles and polymeric nanoparticles require validation of particle-size stability, critical micelle concentration, drug-release profiles and intrapulmonary exposure; clinical benefits should not be inferred solely from in vitro cellular uptake data.

More importantly, translational barriers of delivery platforms should be discussed alongside their pharmacological merits. During large-scale manufacturing of liposomes and micelles, variations in shear force, pressure, pH, temperature, lyophilization conditions and residence time upon batch scale-up may alter particle size, membrane integrity, drug-loading efficiency and release profiles. For this reason, the FDA requires manufacturing processes to demonstrate consistency and reproducibility prior to commercialization and notes that substantial changes in processes, equipment or production sites may demand additional quality assessments and in vivo studies [292]. For polymeric nanoparticles and micelles, batch-to-batch variability can arise from polymer molecular weight and dispersity, drug–carrier interactions, critical micelle concentration, free-drug fraction, and long-term storage stability. In the case of exosomes/EVs, cell sources, culture conditions, isolation and purification procedures, impurity removal, vesicle subpopulation heterogeneity, drug-loading efficiency and potency assays remain non-standardized. Accordingly, they should be regarded as platforms with translational potential rather than mature universal delivery tools [297]. Stimuli-responsive systems further need to prove reproducible response thresholds toward tumor acidity, redox status or enzymatic activity across different patients and lesions; otherwise, stable in vivo release performance may not be achieved.

Therefore, further optimization of delivery systems for lung cancer should not merely pursue more sophisticated carrier designs. Rather, pulmonary deposition and pharmacokinetics, drug-loading efficiency, batch consistency, scalable manufacturing processes, long-term stability, toxicological profiles, regulatory CMC requirements, and clinical practicality should be adopted as joint endpoints. Especially for natural-product-based and drug-repurposing strategies, quantitative correlations among “formulation parameters–lung tissue exposure–target modulation–efficacy/toxicity” should be established at the preclinical stage. Integrated with local drug administration, biomarker-based stratification and rational combination-therapy design, this would build a translational pathway that balances efficacy, safety and manufacturability.

## 7. Clinical Translation: Current Status and Challenges and Discussion

Overall, following the principle of prioritizing clinically relevant evidence chains, this review draws extensively on direct clinical data. Evidence levels are annotated in the corresponding summary table and categorized into four tiers: in vitro, in vivo, clinical trial, and approved clinical use. Notably, substantial discrepancies exist between endpoints adopted in clinical versus preclinical studies. Clinical research primarily focuses on survival-related outcomes, including Overall Survival and Progression-Free Survival, as well as safety metrics such as adverse events (AEs). Accordingly, mechanistic descriptions presented in the table are almost entirely derived from preclinical data.

Upon synthesizing available clinical evidence for various combination regimens, both encouraging and negative signals have been observed. For chemotherapy-based combinations, naturally originating ginsenoside Rg3 and matrine exhibit relatively favorable profiles with promising trends in survival endpoints and safety. Nevertheless, definitive conclusions cannot be drawn due to limited sample sizes, and further research investment is warranted to advance their real-world translational potential. For radiotherapy-combined regimens, β-elemene and nelfinavir show comparatively encouraging performance. Of note, β-elemene emulsion injection is already in clinical use in China. When combined with conventional radiotherapy and chemotherapy, it enhances therapeutic efficacy and alleviates treatment-related toxicities for multiple malignancies, including lung cancer. In contrast, most remaining regimens yield negative outcomes (no obvious therapeutic benefit) or mixed results (co-existence of positive and negative findings). These issues remain to be addressed in future practice, and potential underlying causes are elaborated in the subsequent text.

The journey of compounds from laboratory to market is hampered by multiple confounding factors [68,247,298,299,300], which frequently leads to disappointing outcomes. For instance, despite being a promising candidate for combination therapy, metformin has yielded inconsistent results in real-world clinical trials. Moreover, most compounds exhibiting excellent performance in pre-laboratory studies rarely proceed to subsequent clinical trials and remain merely promising drug candidates. Classified according to drug-development stages, the dilemmas in clinical translation can be addressed by two core questions:

**First, why do preclinical findings with promising in vitro and pre-in vivo performance fail to advance toward clinical application?**

**①. Limitations in pharmacokinetics and tumor tissue distribution.**

Preclinical models often fail to adequately recapitulate human pharmacokinetic profiles, including drug absorption, distribution, renal clearance, insufficient tumor perfusion, and barriers imposed by the tumor microenvironment [299,301]. For example, metformin undergoes minimal hepatic metabolism and is predominantly excreted unchanged via the kidneys; its plasma concentration, intratumoral concentration and duration of exposure are all influenced by renal function and transporter activity [302]. Accordingly, the presence of drug in peripheral blood does not guarantee that concentrations sufficient to inhibit mitochondrial complex I or metabolic pathways have been achieved inside tumor cells. At the methodological level, patient-derived organoids and patient-derived xenograft (PDX) models, along with multi-omics analysis and real-world data, can provide complementary evidence for clinical translation [54,155,270]; among these, organoids and PDX models are particularly suitable for functional drug sensitivity verification and mechanistic studies, while clinical cohorts and real-world data enhance extrapolability and clinical relevance [299,301,303].

**②. Lack of high-quality and reproducible confirmatory data.**

Some preclinical studies rely on single cell lines, small-sample animal experiments or specific experimental conditions, with endpoints mostly limited to cell viability, tumor volume or alterations in molecular markers, which may not predict survival benefits in patients [144,300,304]. Begley and Ellis pointed out that oncological preclinical research suffers from flaws in experimental design, reagent quality, statistical approaches and insufficient independent replication, which compromise result reproducibility and clinical predictive value [305]. Even if a study demonstrates that a drug can inhibit tumor-cell proliferation, such findings should be validated across multiple cell lines, patient-derived models, immunocompetent models and diverse dose-exposure conditions. Results from the Reproducibility Project: Cancer Biology revealed that several high-impact oncological studies could not fully recapitulate the original effect sizes upon replication [306]. Therefore, preclinical “positive” evidence alone may be insufficient to justify progression toward routine clinical practice in the absence of independent validation, pharmacokinetic–pharmacodynamic correlation and well-standardized animal study design.

**Why do promising drugs that have entered clinical trials still fail to achieve satisfactory outcomes?**

**①. Reduced patient adherence and insufficient actual drug exposure.**

The “prescribed dose” in clinical studies is not equivalent to the “actual exposure dose” achieved in patients. Metformin frequently causes gastrointestinal adverse reactions, including nausea, diarrhea, abdominal distension and decreased appetite. Some patients may spontaneously reduce the dose, take medication intermittently, or discontinue treatment, making it impossible to maintain the intended drug exposure [307]. During tumor treatment, chemotherapy-induced nausea, vomiting, appetite loss and poly-drug combination therapy may further impair adherence to oral agents. Insufficient dosing is particularly critical for metabolic-modulating drugs. In the case of metformin, relatively high concentrations are often required in in vitro experiments to exert direct antitumor effects, whereas intratumoral concentrations achieved at human-tolerable doses may not reach such levels [308,309]. Accordingly, unfavorable clinical outcomes do not necessarily imply that the underlying biological mechanism is entirely incorrect; instead, they may reflect a failure to achieve sustained, adequate, tumor-penetrating effective exposure in patients.

**②. Insufficient bioavailability leading to failure to reach preclinically required exposure levels in vivo.**

Many drugs exert potent activity in cell or animal models at concentrations far exceeding the plasma and tumor tissue concentrations achievable following conventional clinical administration. For metformin, in vitro antitumor experiments are commonly performed at millimolar concentrations, while clinically relevant circulating concentrations in humans are only at the micromolar level. This substantial discrepancy between experimentally effective concentrations and clinically achievable concentrations directly impairs translational feasibility [308,310]. Accordingly, robust preclinical efficacy cannot be simply extrapolated to equivalent therapeutic effects in humans. For oral agents, incomplete absorption, first-pass metabolism, gastrointestinal intolerance, and interindividual variability in intestinal transit collectively reduce actual bioavailability. As metformin cellular uptake relies on organic cation transporters, its absorption, distribution and tissue exposure exhibit considerable interpatient heterogeneity [190,302,311]. Consequently, a subset of patients fail to obtain sufficient drug exposure to modulate tumor metabolism or the AMPK/mTOR signaling pathway.

**③. Limited sample size and inadequate statistical power in some clinical studies.**

This issue is frequently observed among the clinical trials discussed in the main text of this review. Early-phase clinical studies are often exploratory by design, characterized by small enrolment sizes, limited endpoint events and insufficient follow-up duration, which renders them incapable of detecting moderate-magnitude therapeutic differences. In settings with low statistical power, true clinical benefits restricted to specific patient subgroups may yield negative overall outcomes owing to inadequate total sample size. As highlighted by Button et al., low statistical power not only elevates the risk of false-negative findings but also undermines the reliability and reproducibility of positive results [312,313].

**④. Drug–drug interactions alter actual drug exposure and therapeutic effects.**

Patients with cancer frequently receive concurrent chemotherapy, targeted therapy, hormonal therapy, antiemetics, analgesics and anti-infective agents. Concomitant multiple-drug administration may therefore affect drug transport, renal clearance or toxicity risk. For instance, renal excretion of metformin depends on transporters, including OCT2, MATE1 and MATE2-K; drugs that inhibit these transporters can modify its clearance rate and elevate drug exposure [302]. This indicates that the same metformin dose may lead to divergent outcomes, such as insufficient drug concentration, excessive concentration or enhanced toxicity under different backgrounds of concomitant medications.

**⑤. Safety concerns may limit dose intensity and the feasibility of combination therapy.**

Safety issues are not merely confined to increased adverse events; they can also indirectly compromise therapeutic efficacy by constraining dosage, delaying treatment, or triggering drug discontinuation. For example, metformin is generally well-tolerated, yet the risk of lactic acidosis rises under conditions such as severe renal impairment, hypoxia, severe infection, dehydration or tissue hypoperfusion. Consequently, treatment suspension or avoidance is often required for such patients [314]. Chemotherapy-related dehydration, infection, renal injury and hepatic dysfunction are common among cancer patients, which may prevent metformin from being administered at the planned dose. When the investigational drug is combined with cytotoxic agents, immunotherapy or targeted therapy, overlapping toxicities and treatment interruptions will further reduce the actual treatment intensity. Hence, the “clinically safe and tolerable dose” is not necessarily equivalent to the “dose capable of exerting antitumor activity”.

**⑥. Insufficient patient stratification masks potential benefit-deriving subgroups.**

Cancer patients exhibit substantial heterogeneity with respect to tumor metabolic profiles, diabetic status, insulin resistance, renal function, prior treatment history, tumor genomic alterations and drug-transporter expression [315,316]. When all patients are enrolled without stratification, small subgroups that could genuinely benefit from metabolic intervention are readily obscured by overall negative trial outcomes. The antitumor effects of metformin may be linked to lowered insulin/IGF signaling, as well as intrinsic metabolic vulnerabilities of tumor cells [317]. Accordingly, uniform treatment response across patients should not be assumed [144,301,318], and patient-subset screening represents an indispensable step to obtaining meaningful results in future studies.

Last but not least, beyond the antitumor synergistic effects, the clinical translation of combination therapies requires careful evaluation of hepatic safety and delivery accessibility. For regimens incorporating natural products or small-molecule agents, particular attention should be paid to their potential modulatory effects on cytochrome P450 enzymes and the consequent drug–drug interactions. For example, the EGFR-TKI osimertinib is contraindicated for co-administration with strong CYP3A inducers, as such combinations may reduce its systemic exposure. This highlights the necessity of assessing CYP3A-associated metabolic risks and dynamically monitoring liver function during combination treatment (https://dailymed.nlm.nih.gov/). In the context of NSCLC brain metastasis, neither the nanoscale dimension nor surface modification confers multi-targeted nanoparticles with intrinsic capacity to efficiently cross the blood–brain barrier (BBB) or blood–tumor barrier (BTB). For instance, pH-responsive paclitaxel nanoformulations achieve enhanced lesion-targeted delivery and antitumor activity in preclinical animal models of NSCLC brain metastasis; nevertheless, such evidence remains confined to preclinical investigations [319]. Accordingly, future studies should further validate the hepatic safety, intracerebral drug distribution, and intracranial therapeutic efficacy of candidate combinatorial regimens without unduly expanding the research scope.

## 8. Conclusions

Overall, natural product research and the repurposing of approved drugs represent two complementary yet pragmatic development pathways in the field of non-small cell lung cancer therapy. Natural products, derived from abundant biological resources, exhibit structural diversity and target a wide range of action mechanisms, often revealing novel modes of action and offering the potential to address existing therapeutic bottlenecks. In contrast, drug repurposing capitalizes on the established clinical safety profiles, shortened development timelines, and high translational efficiency of approved drugs, facilitating rapid advancement to clinical application. This strategy is particularly well-suited to tackle clinical challenges such as the significant heterogeneity and susceptibility to drug resistance observed in non-small cell lung cancer. These two pathways converge significantly in their intervention within the lung cancer survival network—both can modulate tumor proliferation, apoptosis, metabolism, and epigenetic status through a multi-target, multipathway regulatory framework, particularly demonstrating substantial potential for synergistic enhancement in emerging research areas such as ferroptosis, metabolic reprogramming, and epigenetic regulation.

## Figures and Tables

**Table 1:** Classification of signaling pathways.

Functional Category	Signaling Pathway
Driving cell proliferation and survival	PI3K/AKT/mTOR, MAPK/ERK, STAT3
Promoting invasion, metastasis, and therapeutic resistance	Wnt/β-catenin, TGF-β, NF-κB
Regulating metabolism and oxidative stress	Nrf2

**Table 2:** Comparison of advantages and disadvantages between natural products and single-target antitumor drugs.

Type	Advantages	Limitations
Natural product	■ Multi-target synergistic action ■ Chemical structure diversity ■ Broad biological activity spectrum ■ Relatively mild toxicity profile ■ Potential to overcome drug resistance ■ Wide and accessible sources ■ Great scope for derivative optimization ■ Traditional medicine experience support	■ Unclear mechanism of action ■ High druggability challenges ■ Difficulties in quality control ■ High cost of separation and purification ■ High difficulty in total synthesis ■ Insufficient evidence for clinical translation ■ Complex drug–target interactions
Single-target drug	⮚ Well-defined mechanism of action ⮚ High selectivity for the target ⮚ Strict quality control ⮚ Mature synthesis process ⮚ Sufficient clinical evidence ⮚ Controllable drug interactions ⮚ Clear intellectual property ⮚ Defined regulatory approval pathway	⮚ Prone to acquired drug resistance ⮚ Single target ⮚ Difficult to address tumor heterogeneity ⮚ Narrow range of indications ⮚ High R&D cost and long cycle ⮚ Insufficient innovation in chemical scaffolds ⮚ Ignoring network pharmacological effects

## Data Availability

The original data are included in the manuscript, and further queries can be directed to the corresponding author.
